# Safety and Efficacy of Polyethylene Glycol Versus Placebo in the Bowel Preparation for Elective Colorectal Surgeries: A Systemic Review and Meta-Analysis

**DOI:** 10.7759/cureus.81024

**Published:** 2025-03-23

**Authors:** Malak Maaz Hassan, Shafiq Ur Rahman, Malak Bilal Hassan, Taimoor Khan, Intikhab Alam, Atizaz Ahmad, Ata Us Samad, Imran Khan

**Affiliations:** 1 General Surgery, St. Vincent's University Hospital, Dublin, IRL; 2 General Surgery, Saidu Teaching Hospital, Swat, PAK

**Keywords:** colorectal cancer, colorectal cancer surgery, lower gi or colorectal surgery, mechanical bowel preparation (mbp), polyethylene glycol

## Abstract

The most suitable type of preoperative colonic preparation for colorectal surgery is controversial. Polyethylene glycol (PEG) has been widely used and some regard it as more suitable for bowel cleansing. However, it also has some limitations, such as nausea and vomiting. These problems have caused surgeons to question whether bowel cleansing offers any benefit at all.

This study aims to assess the safety and efficacy of PEG, compare it with other available bowel preparations, conduct a detailed analysis of the available evidence, and inform clinical practice guidelines for bowel preparation before elective colorectal surgeries.

MeSH terms and keywords, including "colorectal surgeries", "polyethylene glycol", and "placebo", were used to run a literature search on PubMed, Embase, Cochrane, and Clinicaltrials.gov from inception to January 2025. Randomized controlled trials (RCTs) comparing PEG with placebo for patients undergoing colorectal surgeries were included. Risk ratios (RRs) and 95% confidence intervals (CIs) were pooled using the Mantel-Haenszel method in RevMan (Cochrane Collaboration, London, UK). Random effects meta-analysis was undertaken.

Ten RCTs with a total of 2613 patients were included. Polyethylene showed no significant benefits over placebo regarding quality of bowel preparation (RR = 1.03, 95% CI: 0.91-1.17, p = 0.64) and incidence of surgical site infections (SSIs) (RR = 1.29, 95% CI: 0.95-1.75; p = 0.11). Both groups were comparable in terms of anastomotic leak (RR = 1.14, 95% CI: 0.70-1.85, p = 0.60), intra-abdominal abscess (RR = 0.77, 95% CI: 0.36-1.65, p = 0.50), ileus (RR = 1.16, 95% CI: 0.44-3.05, p = 0.76), anastomotic dehiscence (RR = 0.79, 95% CI: 0.39-1.59, p = 0.51), vomiting (RR = 0.54, 95% CI: 0.27-1.09, p = 0.09), and repeated operations (RR = 0.66, 95% CI: 0.20-2.24, p = 0.51).

PEG has no significant benefits over placebo for bowel preparation before colorectal surgeries. Further research and RCTs are necessary to identify and explore other therapeutic options for patients undergoing colorectal surgeries.

## Introduction and background

Bowel preparation is a crucial step before elective colorectal surgery to reduce the risk of surgical site infections (SSIs) and improve visualization during the procedure [1]. Polyethylene glycol (PEG) is a commonly used bowel preparation agent, which has been shown to be effective in cleansing the bowel [2].

PEG is an osmotic laxative that works by increasing water in the intestines, softening stool, and promoting bowel movement. Upon ingestion, PEG remains in the gastrointestinal tract, drawing water into the intestines through osmosis, which increases the water content and softens the stool. This stimulates intestinal contractions, promotes bowel movements, and clears bowel contents. PEG's mechanism of action is summarized as follows: PEG → osmotic effect → increased water content → softened stool → promoted bowel movements → clearance of bowel contents [3].

However, its safety and efficacy compared to placebo have not been extensively reviewed [4]. Previous studies have reported varying results regarding the safety and efficacy of PEG for bowel preparation, with some studies suggesting that PEG may be associated with adverse events such as nausea, vomiting, and abdominal pain [5]. On the other hand, other studies have found that PEG may be more effective than placebo in achieving adequate bowel cleanliness [6]. The European Society of Gastrointestinal Endoscopy (ESGE) recommends the use of PEG as a bowel preparation agent for colonoscopy [1]. However, the optimal dosage and administration of PEG for bowel preparation are still debated [7]. Furthermore, the use of PEG may be associated with electrolyte imbalance and other adverse events, particularly in patients with underlying medical conditions [8].

The use of PEG in bowel preparation for elective colorectal surgery has been a topic of controversy. While PEG is effective in cleansing the bowel, its use has been associated with adverse events such as nausea, vomiting, and abdominal pain [3]. Additionally, PEG has been linked to electrolyte imbalances and renal impairment in some patients [9]. Furthermore, the effectiveness of PEG in reducing SSIs has been questioned, with some studies suggesting that it may not be as effective as other bowel preparation agents [10]. Therefore, the use of PEG in bowel preparation for elective colorectal surgeries remains a topic of debate among healthcare professionals.

Therefore, a comprehensive systematic review and meta-analysis are necessary to synthesize the existing evidence and provide a definitive conclusion on the safety and efficacy of PEG compared to placebo for bowel preparation before elective colorectal surgery [5]. This review aims to provide a detailed analysis of the available evidence and inform clinical practice guidelines for bowel preparation before elective colorectal surgery [11].

## Review

Methods and methodology

Methods

This meta-analysis was conducted in accordance with the Methodology for Systematic Review of Interventions under the Cochrane Handbook. The findings are reported in accordance with the Preferred Reporting Items for Systematic Reviews and Meta-Analyses (PRISMA) statement. No ethical approval was necessary for this study. The protocol for this review was also submitted to the International Prospective Register of Systematic Reviews (PROSPERO; ID: CRD420251006865).

Data Sources and Searches

Two independent investigators searched the Cochrane Library, MEDLINE/PubMed, and Embase from inception until January 2025, with no date restrictions. Additionally, Clinicaltrials.gov was searched for any ongoing trials. We also manually checked the references of the included randomized controlled trials (RCTs), previously published meta-analyses, and relevant review articles to ensure comprehensive coverage.

The electronic search was conducted using a combination of Medical Subject Headings (MeSH) and the following keywords: (“Polyethylene Glycol” OR “PEG” OR “Bowel Preparation”) AND (“Colorectal Surgery” OR “Elective Colorectal Surgery”) AND (“Placebo” OR “Control”). There were no language restrictions. Full search strategies are provided in Appendix Table A1.

Eligibility Criteria and Outcomes

Inclusion criteria: Studies were included if they were RCTs assessing the efficacy of PEG versus placebo in bowel preparation for elective colorectal surgeries. Additionally, studies had to report at least one of the selected efficacy or safety outcomes and be published as full-text articles in peer-reviewed journals.

Exclusion criteria: Studies were excluded if they were observational studies, case reports, reviews, or conference abstracts. Studies that involved bowel preparation with agents other than PEG or placebo were also excluded. Furthermore, studies that lacked relevant outcome data were not considered for inclusion.

Outcomes: The primary outcome of this analysis was the quality of bowel preparation. Secondary outcomes included SSIs, anastomotic leaks, intra-abdominal collections or abscesses, vomiting, ileus, anastomotic dehiscence or bleeding, abdominal pain and distention, and repeated surgeries.

Data Extraction and Study Selection

EndNote software (Clarivate, Philadelphia, PA) was used to screen all search results for duplicates. Two independent reviewers (S.R. and M.B.H.) screened the titles and abstracts of all articles, followed by full-text assessments for the selected studies. Any discrepancies were resolved through discussion or by consulting the senior author (M.M.H).

Data extraction was performed by two reviewers (T.K. and A.S.) using a standardized Excel sheet (Microsoft Corporation, Redmond, WA), and the senior author confirmed the extracted data. The extracted data included study characteristics (author, year, country, sample size, and patient demographics), intervention details (PEG dosage and administration), comparator details (placebo characteristics), and reported outcomes.

Risk of Bias Assessment

The risk of bias was assessed using the Cochrane Risk of Bias Tool 2 (RoB 2). The methodological quality of the included RCTs was evaluated across five domains: bias due to the randomization process, bias due to deviations from intended interventions, bias due to missing outcome data, bias in the measurement of outcomes, and bias in the selection of reported outcomes. For each study, the risk of bias was categorized as low, high, or having some concerns. Any disagreements were resolved through discussion with a third reviewer (M.M.H).

Publication Bias

To assess potential publication bias, we constructed funnel plots for outcomes, which can be found in the figures presented in the Appendix.

Statistical Analysis

To conduct this meta-analysis, we used Review Manager (RevMan version 5.4.1; Cochrane Collaboration, London, UK) software. Risk ratios (RRs) and 95% confidence intervals were calculated for each outcome. The meta-analysis was performed using a random-effects model with the Mantel-Haenszel method. Pooled estimates were presented using a forest plot, and statistical heterogeneity was assessed using the Higgins I² test.

Sensitivity Analysis

Each study was removed in sequence one by one from the analysis to assess its individual impact on the overall effect. For all outcomes, the exclusion of any single study did not significantly change the results. This suggests that no individual study disproportionately influenced the overall findings. When studies assessed as having a high risk of bias were excluded, the direction and significance of the combined results remained unchanged across all outcomes. For example, the revised RR for SSI after excluding high-risk studies was 1.21 (95% CI: 0.91-1.60), consistent with the primary analysis (RR = 1.29). Similarly, for bowel preparation quality, the RR was 1.06 (95% CI: 0.94-1.20), indicating no significant change in the findings. Re-analysis using a fixed-effects model instead of a random-effects model was performed for outcomes with low heterogeneity. The effect sizes and confidence intervals were nearly identical, confirming that model choice did not significantly affect interpretation. For example, intra-abdominal abscess under the fixed-effects model yielded RR = 0.77 (95% CI: 0.44-1.35), closely matching the random-effects result (RR = 0.77; 95% CI: 0.36-1.65). For main outcomes like SSI and bowel preparation quality, the odds ratio was used to recalculate effect estimates. The statistical significance and direction of effect remained consistent. For example, the odds ratio for SSI was 1.31 (95% CI: 0.93-1.84), closely aligning with the RR-based result. Similarly, the odds ratio for bowel preparation quality was 1.05 (95% CI: 0.88-1.26), mirroring the RR-based finding (RR = 1.03). Overall, the results of the sensitivity analyses support the stability and strength of the main findings. Across all approaches, study exclusion, risk of bias adjustment, model change, and effect measure conversion, the conclusions remained consistent: PEG bowel preparation did not significantly improve bowel cleansing or reduce postoperative complications when compared with placebo.

Results

Study Selection

**Figure 1:**
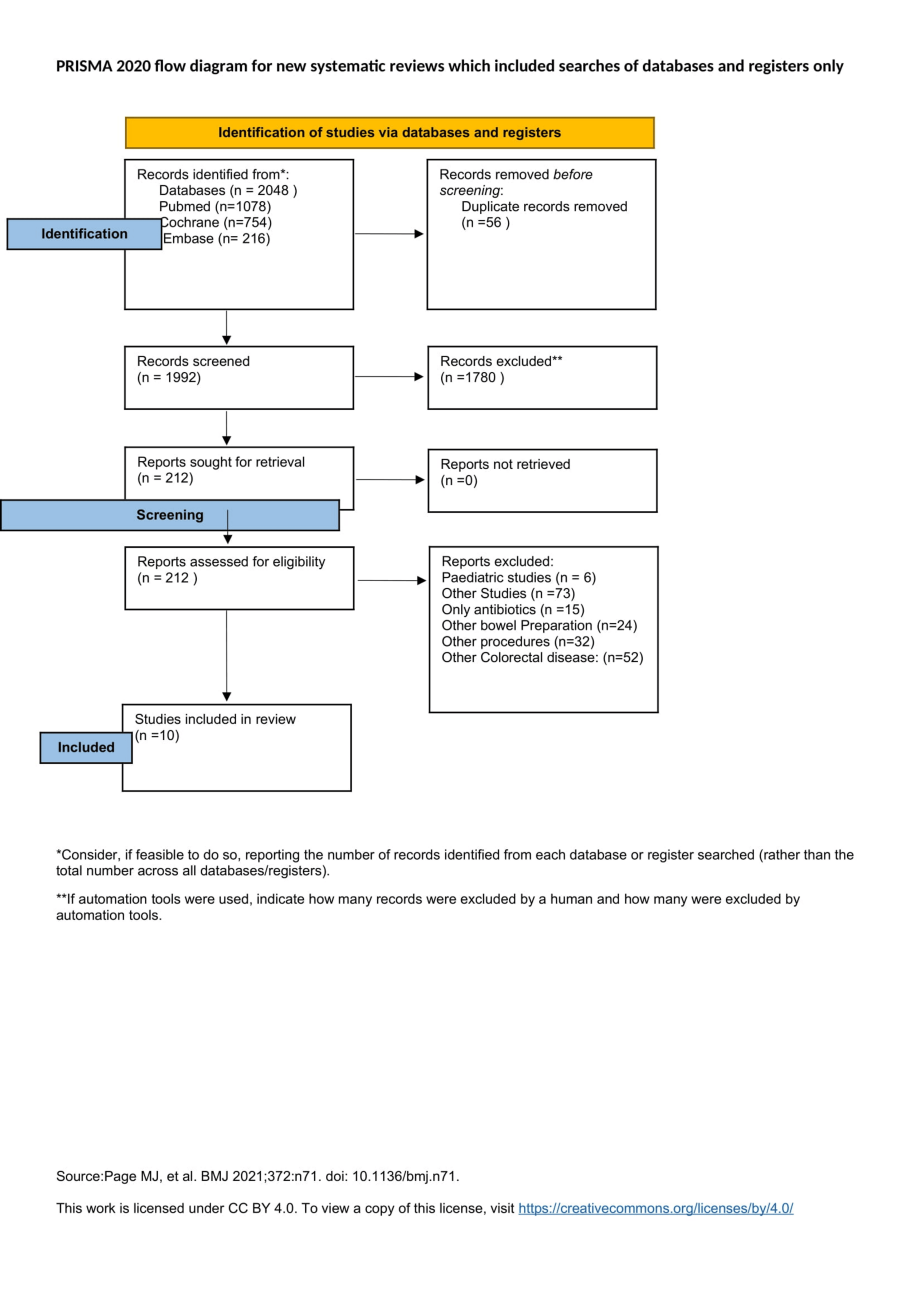
PRISMA flow diagram. PRISMA: Preferred Reporting Items for Systematic Reviews and Meta-Analyses.

The authors extracted 2048 articles via electronically searched databases. A total of 56 duplicate articles were excluded via EndNote software and manual effort. Subsequently, 1992 articles were excluded according to titles and abstracts and 212 articles were completely retrieved and assessed. Ten articles (2613 patients) matching inclusion and exclusion criteria were considered in this meta-analysis (Figure 1).

Study Characteristics

Characteristics of the RCTs are presented in Table 1. Ten studies with a total of 2613 patients were included in the analysis. A total of 1278 patients were given PEG for bowel preparation and 1335 were given placebo. Baseline characteristics such as age, BMI, and gender distribution were comparable between groups. All studies compared PEG with placebo for bowel preparation prior to colorectal surgery.

**Table 1:** Study baseline characteristics. PEG: polyethylene glycol; N.A.: not available.

Study ID	Country	Sample size		Male		Median age		BMI	
		PEG	Placebo	PEG	Placebo	PEG	Placebo	PEG	Placebo
Kristina Žukauskaitė et al. (2024) [12]	Lithuania	18	20	8	12	61 (53-67)	71 (65-77)	28.5 (27.4-29.5)	27.9 (24.7-30.6)
Sodai Arai et al. (2024) [13]	Japan	56	56	34	38	68.0 (56.0-77.0)	66.0 (57.5-74.0)	23.6 (20.9-26.4)	23.7 (21.5-25.6)
Tadashi Yoshida et al. (2023) [14]	Japan	87	86	52	48	65.7 ± 12.1	69.8 ± 10.0	23.1 ± 3.2	23.3 ± 3.8
Bertani E et al. (2011) [15]	Italy	114	115	65	60	63 (28-80)	64 (38-80)	N.A.	N.A.
Kamal MF Itani et al. (2007) [16]	N.A.	303	367	191	175	62.5 (24-94)	59.3 (21-90)	N.A.	N.A.
Platell et al. (2006) [17]	Australia	147	147	95	97	65 (21-90)	66 (22-93)	N.A.	N.A.
Miettinen et al. (2000) [18]	Finland	138	129	68	62	61 (16, 16-89)	64 (16, 21-97)	N.A.	N.A.
Alain Valverde et al. (1999) [19]	N.A.	261	262	132	134	68 ± 13	68 ± 12	N.A.	N.A.
Oliveira et al. (1997) [20]	USA	100	100	47	48	55.5	61.2	N.A.	N.A.
Wolters et al. (1994) [21]	Germany	54	53	30	24	60.2 (27-75)	61.7 (23-75)	N.A.	N.A.

Risk of Bias Assessments

**Figure 2:**
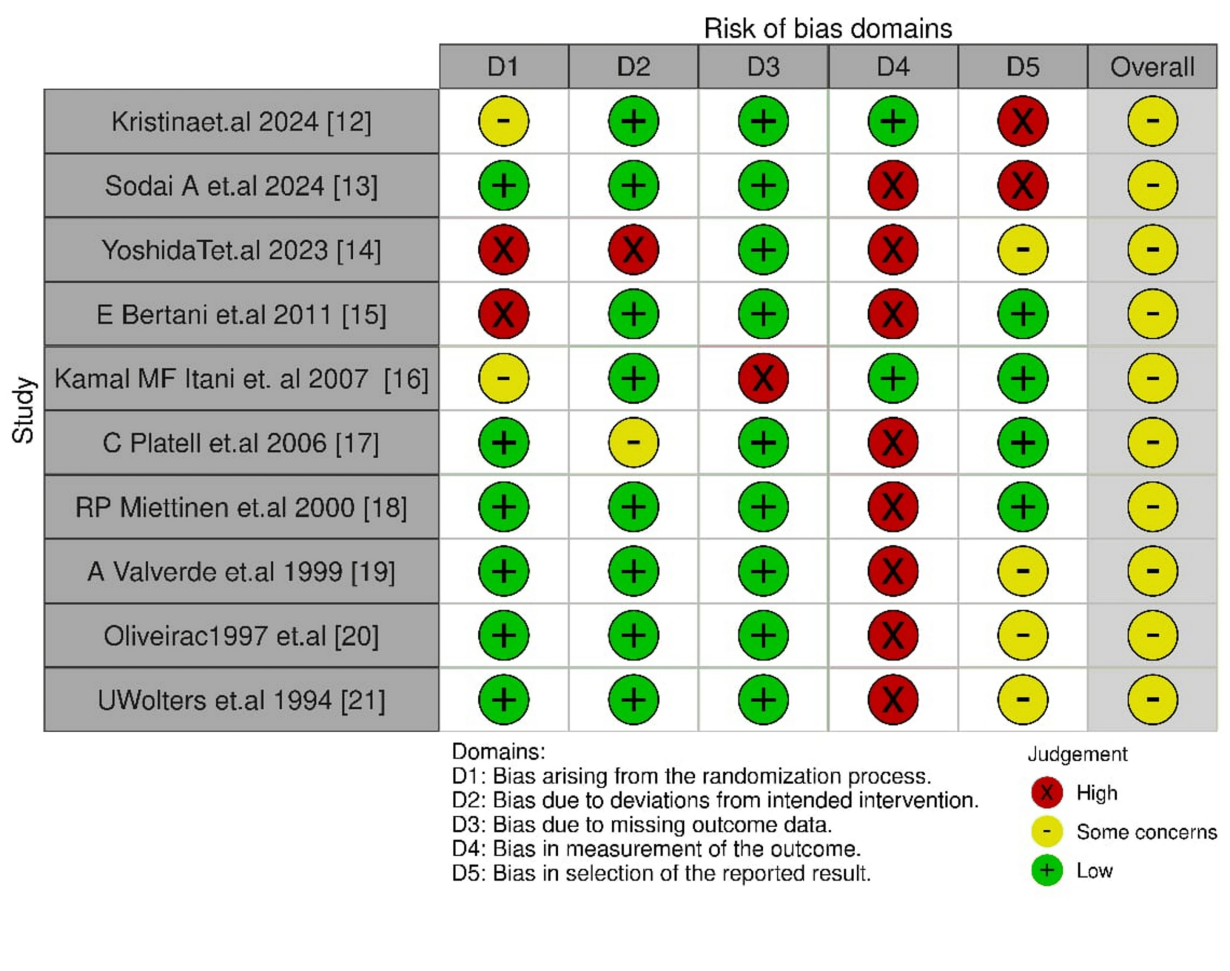
Risk of bias assessment of the randomized controlled trials included in this meta-analysis.

According to the tool from the Cochrane Collaboration, the assessment of risk of bias was conducted over five major domains to assess the reliability of the included studies. The results indicate that most of the studies had a moderate risk of bias, but some had high-risk concerns in certain domains. Missing data and randomization were largely well-controlled, but some studies had high risk in these domains. Outcome measurement and selective reporting were the most frequent biases (Figure 2).

Primary Outcome: Efficacy

**Figure 3:**
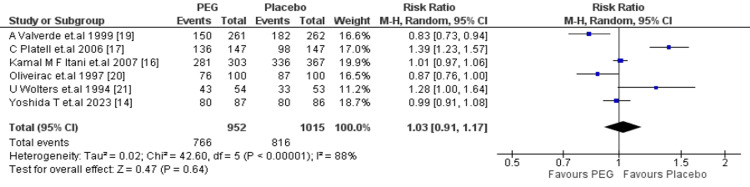
Meta-analysis of bowel preparation. PEG: polyethylene glycol.

Bowel preparation: PEG bowel preparation prior to colorectal surgery was compared with a placebo. Six studies compared PEG with placebo, irrespective of dosage or with the addition of any other adjuvant. From the reported data, PEG did not reveal a significant benefit over placebo regarding quality of bowel preparation (RR = 1.03, 95% CI: 0.91-1.17, p = 0.64 from random effects), with significant heterogeneity between the studies (I^2^ = 88%) (Figure 3).

Secondary Outcomes

**Figure 4:**
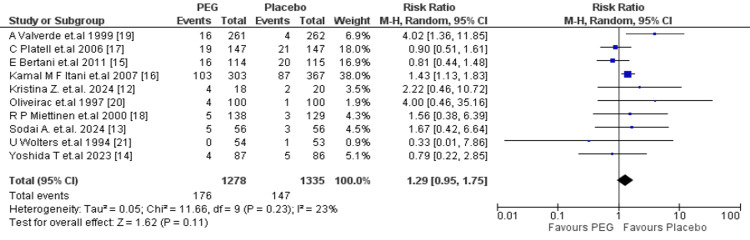
Meta-analysis of surgical site infections. PEG: polyethylene glycol.

Surgical site infection: A total of 10 studies (n = 2613) assessed SSI rates, with PEG (n = 1278) and placebo (n = 1335). The incidence of SSIs was not significantly different between groups with a pool RR of 1.29 (95% CI: 0.95-1.75; p = 0.11). Heterogeneity was low to moderate (I^2 ^= 23%), suggesting minimal variability among studies. A few studies showed increased SSI rates with PEG, whereas others demonstrated decreased risk, exhibiting the disparities probably influenced by differences in surgical techniques and antibiotic administration (Figure 4).

**Figure 5:**
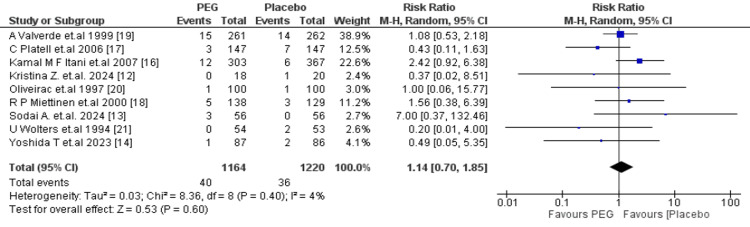
Meta-analysis of anastomotic leak. PEG: polyethylene glycol.

Anastomotic leak: A total of 2384 patients (PEG: 1164; placebo: 1220) were evaluated for anastomotic leaks. The overall RR was 1.14 (95% CI: 0.70-1.85, p = 0.60), showing no statistically significant difference between the two groups. The heterogeneity was low (I^2 ^= 4%, p = 0.40), supporting the credibility of this finding (Figure 5).

**Figure 6:**
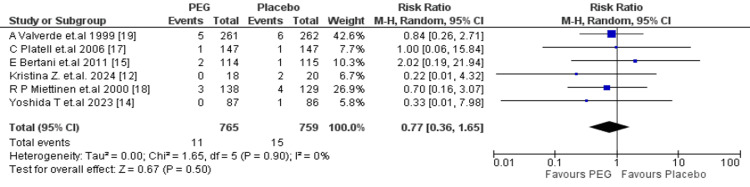
Meta-analysis of intra-abdominal abscess. PEG: polyethylene glycol.

Intra-abdominal abscess: Among 1524 patients (PEG: 765; placebo: 759), pooled RR was 0.77 (95% CI: 0.36-1.65, p = 0.50), which indicated no difference between groups with low heterogeneity (I^2^ = 0%, p = 0.90) (Figure 6).

**Figure 7:**
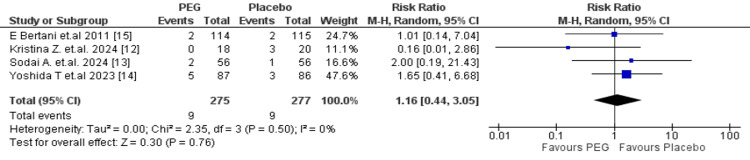
Meta-analysis of ileus. PEG: polyethylene glycol.

Ileus: A total of 552 patients (PEG: 275; placebo: 277) were compared for postoperative ileus. The RR was 1.16 (95% CI: 0.44-3.05, p = 0.76), with low heterogeneity (I² = 0%, p = 0.50), and revealed no difference between the two groups (Figure 7).

**Figure 8:**
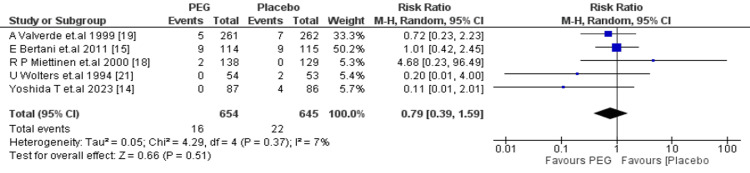
Meta-analysis of anastomotic dehiscence. PEG: polyethylene glycol.

Anastomotic dehiscence: A total of 1299 patients (PEG: 654; placebo: 645) were evaluated for anastomotic dehiscence. The combined RR was 0.79 (95% CI: 0.39-1.59, p = 0.51), which shows no significant difference. Heterogeneity was still low (I² = 7%, p = 0.37) (Figure 8).

**Figure 9:**
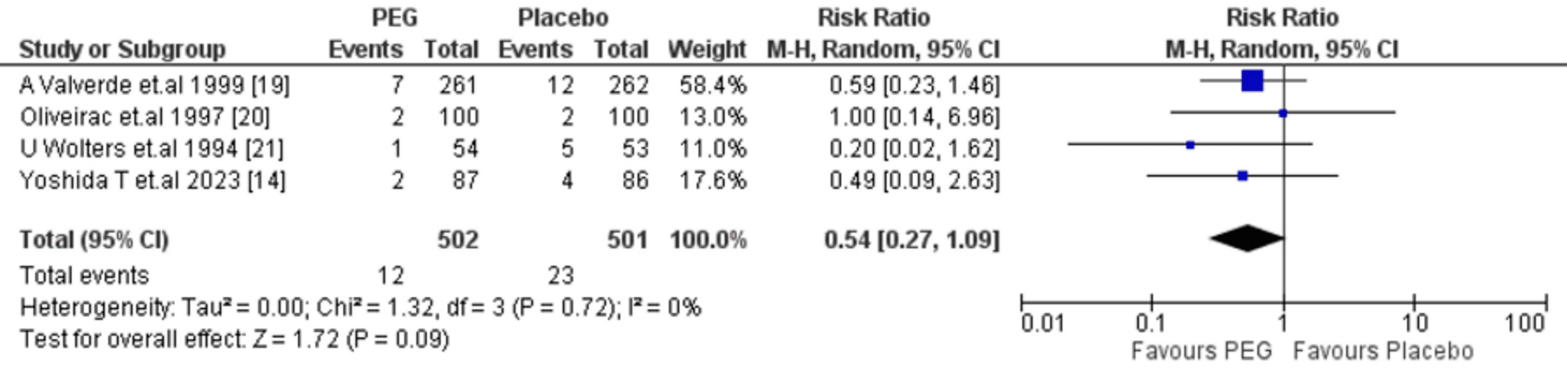
Meta-analysis of vomiting. PEG: polyethylene glycol.

Vomiting: PEG had a possible decrease in vomiting (RR = 0.54, 95% CI: 0.27-1.09), but the finding was not statistically significant (p = 0.09). Low heterogeneity (I² = 0%) indicates consistency among studies (Figure 9).

**Figure 10:**
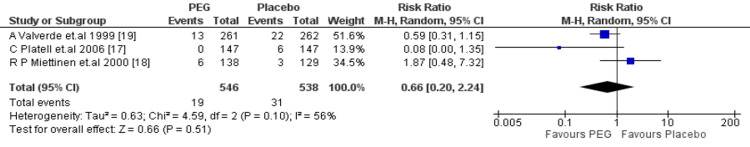
Meta-analysis of repeated operations. PEG: polyethylene glycol.

Repeat operation: The risk of repeat operations was comparable between groups (RR = 0.66, 95% CI: 0.20-2.24, p = 0.51). Moderate heterogeneity (I² = 56%) indicates some study-level variability (Figure 10).

Certainty of Evidence

The certainty of evidence for each outcome was assessed using the GRADE (Grading of Recommendations, Assessment, Development, and Evaluation) approach. Most of the outcomes were rated as low to moderate certainty, mainly due to imprecision, heterogeneity, and potential risk of bias. Table 2 summarizes the key findings and certainty of evidence for each outcome.

**Table 2:** Certainty of evidence of outcomes. RR: risk ratio.

Outcome	Relative effect (95% CI)	No. of participants (studies)	Certainty of evidence	Comments
Adequate bowel preparation	RR = 1.03 (0.91–1.17)	6 studies (n = 1,200)	⬤⬤◯◯ Low	Downgraded for high heterogeneity (I² = 88%) and risk of bias due to unblinded outcome assessment.
Surgical site infection (SSI)	RR = 1.29 (0.95–1.75)	10 studies (n = 2,613)	⬤⬤⬤◯ Moderate	Downgraded for imprecision due to confidence interval crossing the null. Low heterogeneity.
Anastomotic leak	RR = 1.14 (0.70–1.85)	8 studies (n = 2,384)	⬤⬤⬤◯ Moderate	Downgraded for imprecision. Low heterogeneity (I² = 4%) supports reliability.
Intra-abdominal abscess	RR = 0.77 (0.36–1.65)	5 studies (n = 1,524)	⬤⬤◯◯ Low	Downgraded for imprecision and indirectness due to varied definitions and low event rates.
Postoperative ileus	RR = 1.16 (0.44–3.05)	3 studies (n = 552)	⬤⬤◯◯ Low	Downgraded for imprecision and potential subjective assessment of ileus.
Anastomotic dehiscence	RR = 0.79 (0.39–1.59)	4 studies (n = 1,299)	⬤⬤◯◯ Low	Downgraded for imprecision and risk of bias in diagnostic criteria.
Vomiting	RR = 0.54 (0.27–1.09)	3 studies (n = 500)	⬤⬤◯◯ Low	Downgraded for imprecision and subjective reporting. No statistical heterogeneity (I² = 0%).
Repeat operation	RR = 0.66 (0.20–2.24)	4 studies (n = 600)	⬤⬤◯◯ Low	Downgraded for imprecision and inconsistency (I² = 56%). Few events reported.

Discussion

This systematic review and meta-analysis aimed to evaluate the safety and efficacy of PEG compared to other bowel preparations, such as sodium phosphate, magnesium citrate, and low-volume formulations with adjuncts in patients undergoing elective colorectal surgery. Contrary to common assumptions and popular clinical practices, our findings demonstrate no significant benefits of PEG over placebo or alternative regimens in terms of bowel cleansing quality, postoperative outcomes, or overall safety.

The efficacy of PEG in achieving adequate bowel cleansing, often considered its main benefit, was not superior to other mechanical bowel preparations. This finding is also supported by previous studies, for example, Belsey et al., in a meta-analysis of 22 studies involving 3748 participants, found no significant difference between PEG and sodium phosphate (OR = 1.00; 95% CI: 0.67-1.50; p = 0.99) [22]. Similarly, Juluri et al. reported no meaningful differences in 18 RCTs (OR = 1.43; 95% CI: 1.01-2.00) [23], and Kim et al. also found no significant difference in efficacy (one-sided 97.5% CI: -0.5% to 14.3%, p < 0.001) [24]. On the contrary, Tan et al. reported a significantly higher efficacy with sodium phosphate over PEG (OR = 0.75; 95% CI: 0.65-0.88; p = 0.0004) [25], indicating the variability in reported outcomes. These discrepancies may reflect differences in study dosing protocols, populations, or definitions of "adequate" bowel preparation in the trials.

Regarding postoperative complications, PEG did not significantly reduce the incidence of SSIs (RR = 1.29; 95% CI: 0.95-1.75; p = 0.11), consistent with the findings of Zhu et al. (OR = 1.39; 95% CI: 0.85-2.25; P = 0.19) [26] and Tajima et al. [27], who reported similar SSI rates between PEG and other preparations. Rates of anastomotic leakage were also unaffected by PEG use (RR = 1.14; 95% CI: 0.70-1.85; p = 0.60), a trend also observed by Zhu et al., who found a non-significant increase in leak risk associated with PEG (OR = 1.78; 95% CI: 0.95-3.33; p = 0.07) [26]. Furthermore, no statistically significant differences were found in the rates of intra-abdominal abscess formation (RR = 0.77; 95% CI: 0.36-1.65; p = 0.50) or anastomotic dehiscence (RR = 0.79; 95% CI: 0.39-1.59; p = 0.51).

Other peri-operative outcomes, including vomiting (RR = 0.54; 95% CI: 0.27-1.09; p = 0.09), postoperative ileus (RR = 1.16; 95% CI: 0.44-3.05; p = 0.76), and the need for reoperation (RR = 0.66; 95% CI: 0.20-2.24; p = 0.51), also showed no significant differences between PEG and comparator regimens. These findings are supported by the results of Kim et al. [24] and Sun et al. [28], who reported no significant differences in tolerability or rates of adverse effects. Overall, the evidence shows that PEG offers no significant advantage over other bowel preparations in reducing postoperative complications.

These results question the established justification for routine use of PEG in elective colorectal surgeries, particularly in the context of Enhanced Recovery After Surgery (ERAS) protocols. From a safety point of view, while PEG did not increase the risk of adverse events, it also did not show a clear benefit over other preparations. In addition, patient tolerability remains an issue with PEG due to the large volume required, which may impact compliance and effectiveness, particularly in frail elderly patients, indicating that the choice of regimen may be guided more by patient tolerability, preference, and safety profiles rather than efficacy alone.

Using the GRADE approach, we found that the certainty of evidence for SSIs and anastomotic leaks was moderate, while most of the other outcomes were rated as low. It is because of imprecision, heterogeneity, and subjective assessment. These ratings highlight the need for more standardized trials in the future.

Besides the merits, this meta-analysis has some limitations as well. First, inconsistency in the timing of bowel preparation relative to surgery may have affected outcomes. Second, adherence to PEG was inadequate in some studies due to its high volume and unfavorable taste, potentially compromising bowel preparation quality. Third, heterogeneity in patient characteristics such as age, co-morbidities, and oncologic treatments may have influenced postoperative complications.

## Conclusions

This meta-analysis showed no significant benefits of PEG compared to placebo for bowel preparation before colorectal surgery. Despite its common use, PEG did not demonstrate a statistically significant improvement in bowel preparation, SSI, anastomotic leak, ileus, or intra-abdominal collection across the included studies. Although some studies have shown that PEG has minor benefits over placebo, the overall analysis does not support these findings. In addition, the adverse effects were almost the same in both groups, suggesting no superiority of PEG as a treatment. These findings suggest that further research and RCTs are necessary to identify and explore alternative therapeutic options for patients undergoing colorectal surgery.

## Figures and Tables

**Figure 11:**
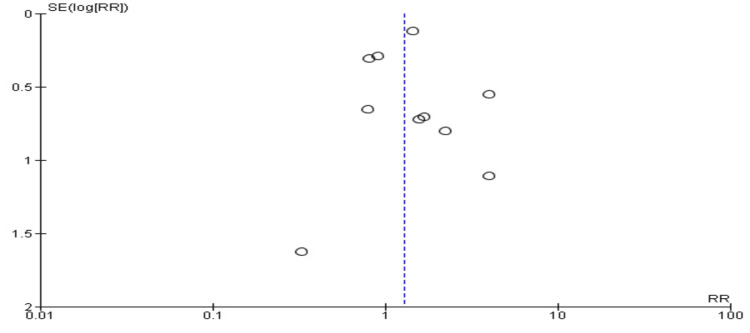
Funnel plot of meta-analysis of surgical site infections.

**Figure 12:**
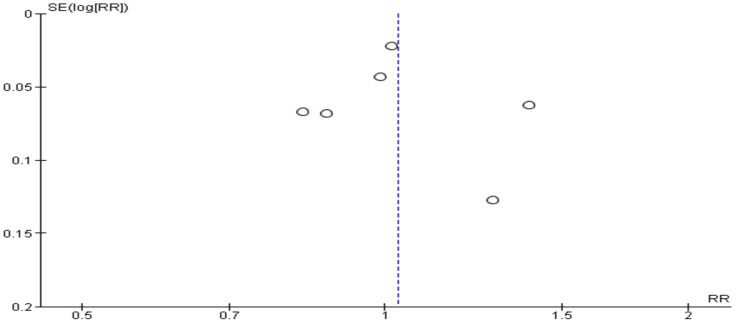
Funnel plot of meta-analysis of bowel preparation.

**Figure 13:**
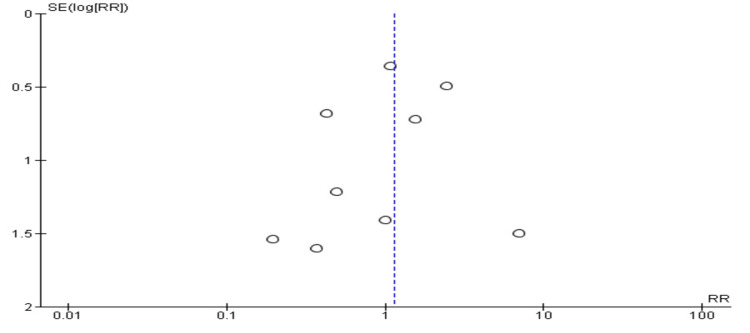
Funnel plot of meta-analysis of anastomotic leak.

**Figure 14:**
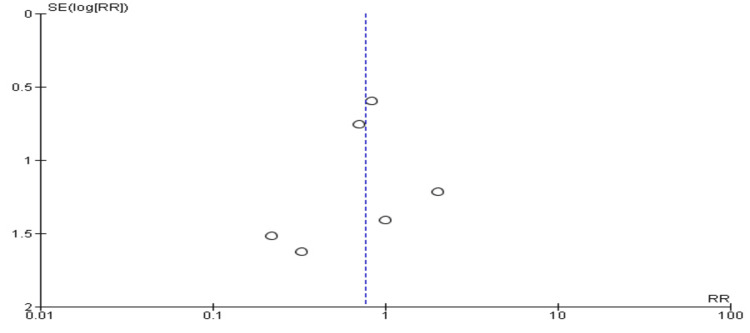
Funnel plot of meta-analysis of intra-abdominal abscess.

**Figure 15:**
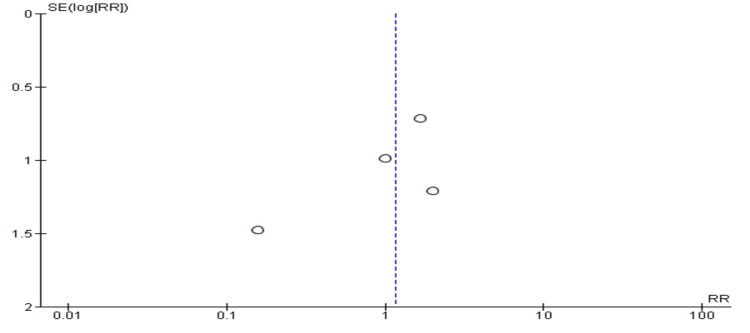
Funnel plot of meta-analysis of ileus.

**Figure 16:**
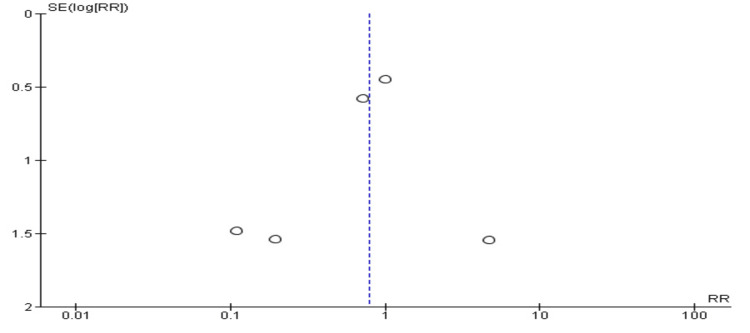
Funnel plot of meta-analysis of anastomotic dehiscence.

**Figure 17:**
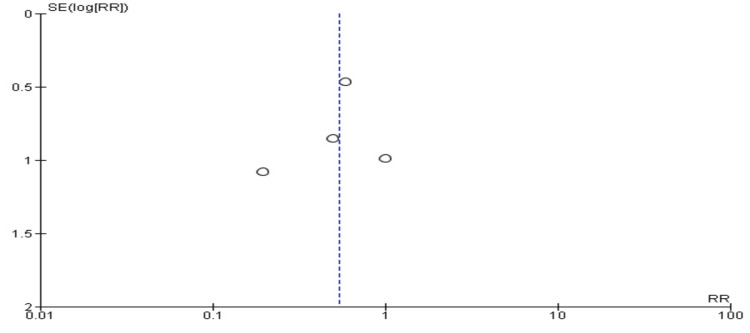
Funnel plot of meta-analysis of vomiting.

**Figure 18:**
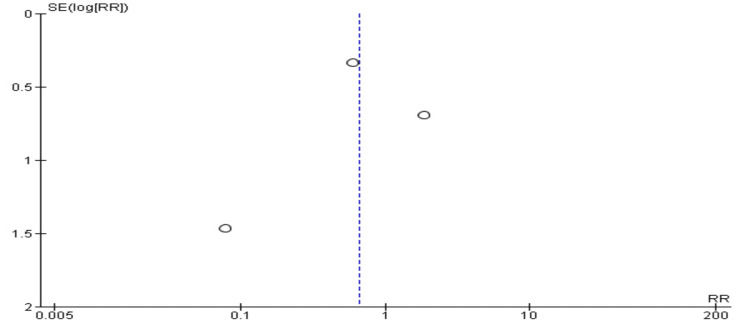
Funnel plot of meta-analysis of repeated operation.

## Appendices

Funnel plots

Table A1

**Table 3:** Search string of each database.

Database	Search string
PubMed (MEDLINE)	((((((((((("colorectal surgery"[MeSH Terms]) OR (Colon and Rectal Surgery Specialty)) OR (Surgery Specialty, Colon and Rectal)) OR (Surgery, Colorectal)) OR (Colon Surgery Specialty)) OR (Specialty, Colon Surgery)) OR (Surgery Specialty, Colon)) OR (Proctology)) OR (Rectal Surgery Specialty)) OR (Specialty, Rectal Surgery)) OR (Surgery Specialty, Rectal)) AND (((((((((("intestines"[MeSH Terms]) OR (Cathartic)) OR (Bowel Evacuant)) OR (Evacuant, Bowel)) OR (Purgative)) OR (Bowel Evacuants)) OR (Evacuants, Bowel)) OR (Purgatives)) OR (Bowel Preparation Solutions)) OR (Bowel Preparation Solution))
Embase	('polyethylene glycol'/exp OR 'polyethylene glycol':ti,ab OR peg:ti,ab) AND ('placebo'/exp OR placebo:ti,ab OR control:ti,ab) AND ('bowel preparation'/exp OR 'mechanical bowel preparation':ti,ab OR 'bowel cleansing':ti,ab) AND ('colorectal surgery'/exp OR 'elective colorectal surgery':ti,ab) AND (randomized controlled trial/exp OR randomized:ti,ab OR trial:ti,ab)
Cochrane Central	(polyethylene glycol OR PEG) AND (placebo OR control) AND ("bowel preparation" OR "mechanical bowel preparation" OR "bowel cleansing") AND ("colorectal surgery" OR "elective colorectal surgery") AND (randomized OR trial)
